# Supplementary material for: Human mtRF1 terminates COX1 translation and its ablation induces mitochondrial ribosome-associated quality control
Source: Nat Commun. 2022 Oct 27;13:6406. doi: 10.1038/s41467-022-34088-w (PMC9613700; doi:10.1038/s41467-022-34088-w)
Supplement: Supplementary file 1 — Supplementary Information [file 41467_2022_34088_MOESM1_ESM.pdf]

Human mtRF1 terminates COX1 translation and its ablation induces mitochondrial ribosome-associated quality control

Franziska Nadler[1], Elena Lavdovskaia[1,2], Angelique Krempler[1], Luis Daniel Cruz-Zaragoza[1], Sven Dennerlein[1] and Ricarda Richter-Dennerlein[1,2,3*]

[1]Department of Cellular Biochemistry, University Medical Center Goettingen, D-37073 Goettingen, Germany. [2]Cluster of Excellence "Multiscale Bioimaging: from Molecular Machines to Networks of Excitable Cells" (MBExC), University of Goettingen, D-37075 Goettingen, Germany. [3]Goettingen Center for Molecular Biosciences, University of Goettingen, D-37077 Goettingen, Germany.

[*] *correspondence: ricarda.richter@med.uni-goettingen.de*

Supplementary information

Supplementary Figures

```
                          α5-helix                           PxT                          GGQ
                        ---------->
T.thermophilus RF1   ...VEIRAG--TGGEEAALFARDLFNMYLRF...ESGGHRVQRVPVT---ETQGRIHTSTA...IRIDVMRASGPGGQGVNTTDS...
        E.coli RF1   ...LEVRAG--TGGDEAALFAGRLFRMYSRY...ESGGHRVQRVPAT---ESQGRIHTSAC...LRIDTFRSSGAGGQHVNTTDS...
            mtRF1a   ...LEVTAG--VGGQEAMLFTSEIFDMYQQY...EGGVHRVQRVPKT---EKQGRVHTSTM...LRIDTKRASGAGGQHVNTTDS...
             mtRF1   ...LEVTAGRTTGGDICQQFTREIFDMYQNY...EGGIHRVQRIPEVGLSSRMQRIHTGTM...LRIDTFRAKGAGGQHVNKTDS...
        ICT1 (mL62)  ...LLPPPAR-----------CPRRALHKQK...---------------------------...LTISYCRSSGPGGQNVNKVNS...
          C12ORF65   ...-MSTVG---------LFH---FPTPLTR...---------------------------...LEEQFVKGHGPGGQATNKTSN...
```

Supplementary Fig. 1. Sequence alignment of mitochondrial and bacterial release factors. Extended alignment as shown in Fig. 1a is provided with the decoding motifs labeled in green and blue and the GGQ motif in red.

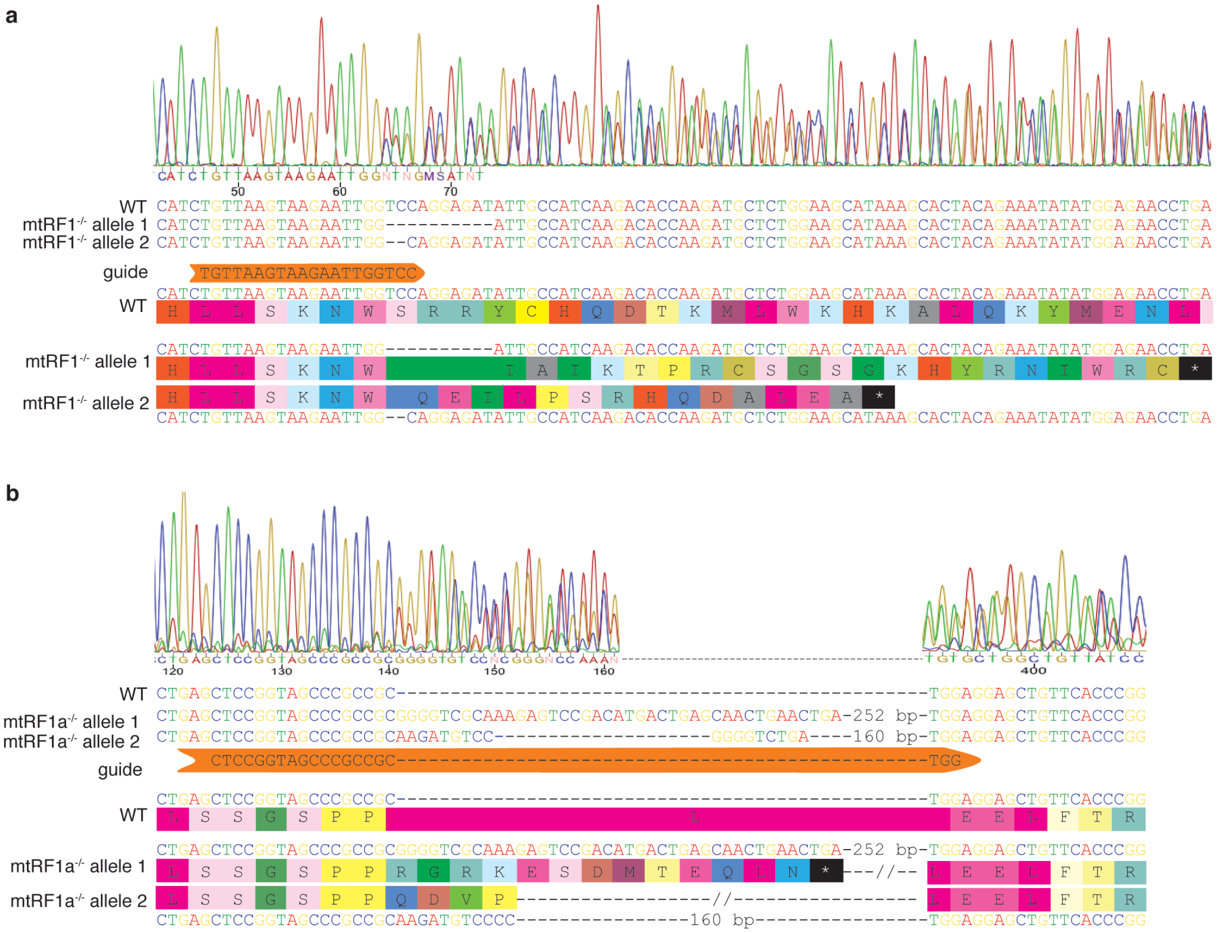

Supplementary Fig. 2. Genomic sequences of *mtRF1*$^{-/-}$ (a) and *mtRF1a*$^{-/-}$ (b).
Genomic DNA was isolated from the respective knockout cell lines, respective target region was PCR amplified and products were analyzed using TOPO cloning and subsequent sequencing. Chromatograms and respective DNA sequences of allele 1 and 2 with corresponding amino acid sequences are provided.
Source data are provided as a Source Data file.

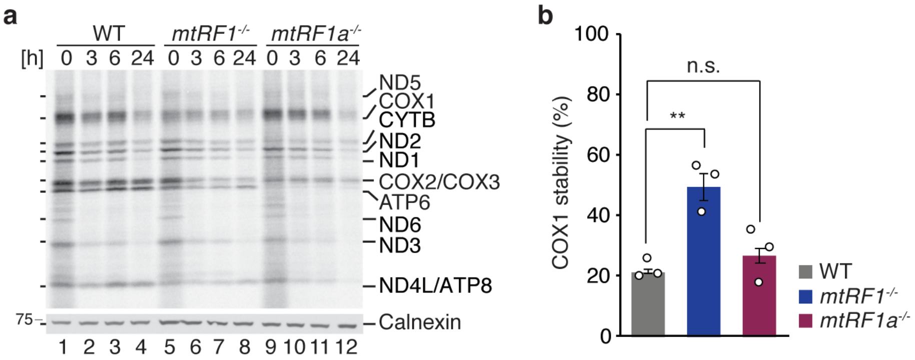

Supplementary Fig. 3. Stability of newly synthesized COX1. a) Mitochondrial translation products were labelled by [^{35}S]Methionine incorporation for 1h in anisomycin treated cells. Radioactive media was replaced for the indicated time points and samples were analysed by western blotting and autoradiography. b) COX1 protein levels were quantified after 24h chase and are presented as percentage of the starting point (t = 0h) in the respective cell lines. Individual data points are shown as circles Statistical analysis was carried out as two-sample (equal variances) one-tailed Student's t-test with n = 3 biologically independent samples and shown as mean ± SEM. Significance was defined as $p \leq 0.01$ ** and $p > 0.05$ as not significant (n.s.). Source data are provided as a Source Data file.

a COX1 termination codons

```
Homo sapiens
5‘...CCACCCUACCACACAUUCGAAGAACCCGUAUACAUAAAAUCUAGACAAAAAAGGAAGGAAUCGAA...3‘
  ... P  P  Y  H  T  F  E  E  P  V  Y  M  K  S  *

Pan troglodytes
5‘...CCACCCUACCACACAUUCGAAGAACCCGUAUACAUAAAAUCUAGACAAAAAAGGAAGGAAUCGAA...3‘
  ... P  P  Y  H  T  F  E  E  P  V  Y  M  K  S  *

Mus musculus
5‘...CCAUAUCACACAUUCGAGGAACCAACCUAUGUAAAAGUAAAAUAAGAAAGGAAGGAAUCGAACCC...3‘
  ... P  Y  H  T  F  E  E  P  T  Y  V  K  V  K  *

Rattus norvegicus
5‘...CCCUACCACACAUUCGAAGAACCUUCCUAUGUAAAAGUUAAAUAAGAAAGGAAGGAUUCGAACCC...3‘
  ... P  Y  H  T  F  E  E  P  S  Y  V  K  V  K  *

Bos taurus
5‘...CCAUAUCACACAUUUGAAGAACCCACCUAUGUUAACCUAAAAUAAGAAAGGAAGGAAUCGAACCC...3‘
  ... P  Y  H  T  F  E  E  P  T  Y  V  N  L  K  *

Sus scrofa
5‘...CCCUAUCACACAUUUGAAGAACCAACAUAUAUCAACCUAAAAUAAGCAUAAGAAAGGAAGGAAUC...3‘
  ... P  Y  H  T  F  E  E  P  T  Y  I  N  L  K  *
```

b ND6 termination codons

```
Homo sapiens
5‘…UUUGUUGGUGUAUAUAUUGUAAUUGAGAUUGCUCGGGGGAAUAGGUUAUGUGAUUAGGAGUAGGG…3‘
  … F  V  G  V  Y  I  V  I  E  I  A  R  G  N  *

Pan troglodytes
5‘…UUUGUUGGUGUAUACAUUGUGAUUGAGAUUGCUCGGGGGAAUAGGUUAUGUGAUUAGGAGUAAGG…3‘
  … F  V  G  V  Y  I  V  I  E  I  A  R  G  N  *

Mus musculus
5‘…UUGUUUGCGGGUAUUUUUAUUAUUAUCGAGAUUACUCGAGAUUAAUUGAGUAUAAGAUAAUAAUU…3‘
  … L  F  A  G  I  F  I  I  I  E  I  T  R  D  *

Rattus norvegicus
5‘…UUGUUUGCGGGGAUUUUUAUUAUUAUUGAAAUCACUCGGGAUUAAGUGUGUGUAAGAUAAAAAUU…3‘
  … L  F  A  G  I  F  I  I  I  E  I  T  R  D  *

Bos taurus
5‘…UUAAUUGGUGUUGUGGUUAUUAUAGAAAUUACUCGUGGAAAUUAAAUAAGAUUAUGCUGAUAAGG…3‘
  … L  I  G  V  V  V  I  M  E  I  T  R  G  N  *

Sus scrofa
5‘…CUUAUUGGAGUUGUUGUUAUUAUAGAGAUUACUCGUGGUAAUUAAAUAGUGUUAUGCUGAUUAUA…3‘
  … L  I  G  V  V  V  I  M  E  I  T  R  G  N  *
```

Supplementary Fig. 4. Comparison of COX1 (a) and ND6 (b) termination codons in different species. Only the 3’ end for COX1 and ND6 mRNA including the UTRs are presented with the corresponding amino acid sequence. Termination codons are marked in bold.

```
mtRF1 M.musculus        -MSHHLCIWLFRNP-FLRACPQRHVFLSCQQFRQISLDTRPWNFRQKKTHVLYQLLNKSW  58
mtRF1 R.norvegicus      -MSFHLCVWLFRNLSSLSACSQRHVFLYGQQFRQINLDPRLWNFRQNKTHVLYRLLNKSW  59
mtRF1 H.sapiens         -MNRHLCVWLFRHPSL-NGYLQCHIQLHSHQFRQIHLDTRLQVFRQNRNCILH-LLSKNW  57
mtRF1 B.taurus          MNRRHLFAWLFRHLSL-NGHLQCHVHRHSHQLTQIPLDTRLWVFRRNRNHTVHRLLNKNC  59
mtRF1a M.musculus       -----------------------------------------------------------M  1
mtRF1a R.norvegicus     -----------------------------------------------------------M  1
mtRF1a H.sapiens        -----------------------------------------------------------M  1
mtRF1a B.taurus         -----------------------------------------------------------M  1

mtRF1 M.musculus        SRGCCHQGTRKLWKHKALQKYMEDLNKEYQTLDQCLQGISENEGDRR------------A  106
mtRF1 R.norvegicus      SRGYC-QGTRKLWKHKALQKYMEGLNDEYQSLDQCLQDISEDEGDRR------------A  106
mtRF1 H.sapiens         SRRYCHQDTKMLWKHKALQKYMENLSKEYQTLEQCLQHIPVNEENRR------------S  105
mtRF1 B.taurus          SRRYCHQDTSMLWKHKALQKYMEDLNKEYQTLDHCLHHISASEGDRR------------S  107
mtRF1a M.musculus       RSGFLS-GARRLWARR-------AFSRTPPPSEELL----ARGGPLRAFLERRVGSEAGG  49
mtRF1a R.norvegicus     RSGFLR-SARRLWARR-------AISRMPPPSEELL----ARGGPLRAFLERRVGSEAGG  49
mtRF1a H.sapiens        RSRVLWGAARWLWPRRAVGPARRPLSSGSPPLEELF----TRGGPLRTFLERQAGSEAH-  56
mtRF1a B.taurus         RPRLVWNVFRGFWARRGVVPACRHLSCSNLPLEELF----ARGGALRTFLERQVGAEAQ-  56
                                   :* ::        :.       :. :         *

mtRF1 M.musculus        LHRRHAQLAPLAAVYQEIQEAEQAIEELESLCKSLNKQDEKQLQELVSEERQIIDQKIHR  166
mtRF1 R.norvegicus      SHRRHAELAPLVAVYQEIQEAEQAIEELESMCKSLNKQDEKQLQELVSEERQIIDQKIHR  166
mtRF1 H.sapiens         LNRRHAELAPLAAIYQEIQETEQAIEELESMCKSLNKQDEKQLQELALEERQTIDQKINM  165
mtRF1 B.taurus          LTRRHAELAPLAVIYKEIQEAEQAIEELESMCKSLNKQDEKQLQELALEERQTIAQKINM  167
mtRF1a M.musculus       LDAGYPQ---LAAAARLLSEKERELRDTESLLH----DENEDLKKLAESEIALCQKQITE  102
mtRF1a R.norvegicus     LDAGSPQ---LAAAARLLNEKERELRDTESLLH----DENEDLKKLAESEIALCQKEIAE  102
mtRF1a H.sapiens        LKVRRPE---LLAVIKLLNEKERELRETEHLLH----DENEDLRKLAENEITLCQKEITQ  109
mtRF1a B.taurus         FQVRRPE---LVAVAKLLSDKEQELQETQHLLH----DENEDLRKLAENEITSCEKEIAQ  109
                              :   * .  : :.: *: :.: : : :    ::::*::*. .*     ::*

mtRF1 M.musculus        LYSELLERLVPKEKYDWSDVILEVTSGRTTGGDICQQFTREIFDMYQNYSYYKHWKFELL  226
mtRF1 R.norvegicus      LYSELLEHLVPKEKCDRSNVILEVTSGRTTGGDICQQFTREIFDMYQNYSYYKHWRFELL  226
mtRF1 H.sapiens         LYNELFQSLVPKEKYDKNDVILEVTAGRTTGGDICQQFTREIFDMYQNYSCYKHWQFELL  225
mtRF1 B.taurus          LYSELFQSLLPKEKYDKNDVILEVTSGRTTGGDICQQFTREIFDMYQNYSSYKHWRFELL  227
mtRF1a M.musculus       LKHQIISLLVPSEEMDGSDLILEVTAG--VGGQEAMLFTSEMFDMYQQYAAFKRWHFETL  160
mtRF1a R.norvegicus     LKHRIISLLVPSEDMDGSDLILEVTAG--VGGQEAMLFTSEMFDMYQQYAAFKRWHFETL  160
mtRF1a H.sapiens        LKHQIILLLVPSEETDENDLILEVTAG--VGGQEAMLFTSEIFDMYQQYAAFKRWHFETL  167
mtRF1a B.taurus         LKHQIILLLVPSEETDKNDLILEVTAG--VGGQEAMLFTSEIFDMYQQYAAFKRWHFETL  167
                        *  .::  *:*.*. * .::*****:*  .**: .  ** *:*****:*: :*:*:** *

mtRF1 M.musculus        NYTPADYGGLHHAAARISGDSVYKHLKYEGGIHRVQRIPEVGLSSRMQRIHTGTMSVIVL  286
mtRF1 R.norvegicus      NYTPADYGGLHHAAARISGDSVYKHLKYEGGIHRVQRIPEVGLSSRMQRIHTGTMSVIVL  286
mtRF1 H.sapiens         NYTPADYGGLHHAAARISGDGVYKHLKYEGGIHRVQRIPEVGLSSRMQRIHTGTMSVIVL  285
mtRF1 B.taurus          NYTPADYGGLHHAAARISGDNVYKHLKYEGGIHRVQRIPEVGLSSRMQRIHTGTMSVIVL  287
mtRF1a M.musculus       EYFPSELGGLRHASASVGGPEAYRHMKFEGGVHRVQRVPKTE---KQGRIHTSTMTVAIL  217
mtRF1a R.norvegicus     EYFPSELGGLRHASASIGGPEAYRHMKFEGGVHRVQRVPKTE---RQGRIHTSTMTVAIL  217
mtRF1a H.sapiens        EYFPSELGGLRHASASIGGSEAYRHMKFEGGVHRVQRVPKTE---KQGRVHTSTMTVAIL  224
mtRF1a B.taurus         EYFPSEIGGLRHASASIGGSEAYKHMKFEGGVHRVQRVPKTE---KQGRIHTSTMTVAIL  224
                        :* *:: ***:**:* :.*  .*:*:*:***:*****:*:.     :  *:**.**:* :*

mtRF1 M.musculus        PQPDEVDVKVDPKDLRVDTFRARGAGGQHVNTTDSAVRLVHIPTGLVVECQQERSQLKNK  346
mtRF1 R.norvegicus      PQPDEVDVKVDPRDLRVDTFRARGAGGQHVNTTDSAVRLVHIPTGLVVECQQERSQLKNK  346
mtRF1 H.sapiens         PQPDEVDVKLDPKDLRIDTFRAKGAGGQHVNKTDSAVRLVHIPTGLVVECQQERSQIKNK  345
mtRF1 B.taurus          PHPDEVDVKVDPKDLRIDTFRAKGAGGQHVNTTDSAVRLVHIPTGLVVECQQERSQIKNK  347
mtRF1a M.musculus       PQPTEIKLVINPKDLRIDTKRASGAGGQHVNTTDSAVRIVHLPTGIISECQQERSQLKNR  277
mtRF1a R.norvegicus     PQPTEIKLVINPKDLRIDTKRASGAGGQHVNTTDSAVRIVHLPTGIISECQQERSQLKNR  277
mtRF1a H.sapiens        PQPTEINLVINPKDLRIDTKRASGAGGQHVNTTDSAVRIVHLPTGVVSECQQERSQLKNK  284
mtRF1a B.taurus         PQPTEINLVINPKDLRIDTKRASGAGGQHVNTTDSAVRIVHLPTGIVSECQQERSQLKNK  284
                        *:* *:.: ::*:***:** **********.******:**:***:: ********:**:

mtRF1 M.musculus        EIALRVLRARLYQQIIEKDRCQQQNARKLQVGTRAQSERIRTYNFTQDRVTDHRIAYEVR  406
mtRF1 R.norvegicus      EIALRVLRARLYQQIIEKDKCQQQNARKLQVGTRAQSERIRTYNFTQDRVTDHRIAYEVR  406
mtRF1 H.sapiens         EIAFRVLRARLYQQIIEKDKRQQQSARKLQVGTRAQSERIRTYNFTQDRVSDHRIAYEVR  405
mtRF1 B.taurus          EIALRVLRARLYQQIIEKDKCQQRSARKLQVGTRAQSERIRTYNFTQDRVTDHRIAYEVR  407
mtRF1a M.musculus       ELAMKKLRARLYSMHLEEETAKRYNARKIQVGTKGRSEKIRTYNFPQNRVTDHRINKSLH  337
mtRF1a R.norvegicus     ELAMKKLRARLYSMRLEEETAKRYSARKIQVGTKGRSEKIRTYNFPQNRVTDHRINKSLH  337
mtRF1a H.sapiens        ELAMTKLRAKLYSMHLEEEINKRQNARKIQIGSKGRSEKIRTYNFPQNRVTDHRINKTLH  344
mtRF1a B.taurus         EMAMKKLRAKLYSLQLEEETSKRYNARKIQIGTKGRSEKIRTYNFPQNRVTDHRINKSLH  344
                        *:*:  ***:**.  :*::  :: .***:*:*::.:**:****** *:**:****    ::

mtRF1 M.musculus        DIKEFLRGEKCLDQLIERLLQSADEEAISEFLDESLQSVK   446
mtRF1 R.norvegicus      DIKEFLRGEKCLDQLIERLLQSADEEAIAEFLDESLQSVK   446
mtRF1 H.sapiens         DIKEFLCGGKGLDQLIQRLLQSADEEAIAELLDEHLKSAK   445
mtRF1 B.taurus          NIKEFLCGEKCLDQLIQRLLQSADEEAITEFLDENLKSVK   447
mtRF1a M.musculus       DLESFMQGDCLLDDMIQSLKDCSDYEALVEMISRRD----   373
mtRF1a R.norvegicus     DLESFMQGDCLLDDLIQSLKDYSDYESLVEMISRKD----   373
mtRF1a H.sapiens        DLETFMQGDYLLDELVQSLKEYADYESLVEIISQKV----   380
mtRF1a B.taurus         DLETFMQGEYLLDELVQSLKDYANYESLVEIIAKEV----   380
                        ::: *: *    **:::: * : :: *:: *:: .
```

Supplementary Fig. 5. CLUSTAL multiple sequence alignment of mtRF1 and mtRF1a.

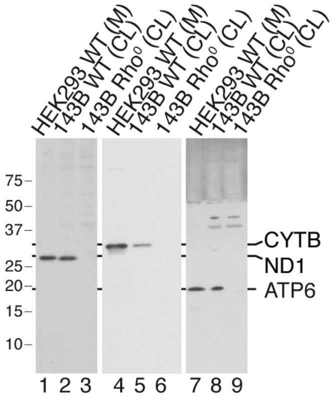

Supplementary Fig. 6. Antibody validation. Isolated mitochondria (M) from HEK293 cells, and cell lysates (CL) from 143B wildtype (WT) and Rho^0 cells were subjected to SDS-PAGE followed by western blotting. Antibodies against ND1 (lane 1-3), CYTB (4-6) and ATP6 (7-9) were applied as indicated. Antibodies were tested once using Rho0 cells and further confirmed by using *mtRF1a*$^{-/-}$ cells. Source data are provided as a Source Data file.

Supplementary Table 1. Key reagents

REAGENT or RESOURCE	SOURCE	IDENTIFIER
**Antibodies**		
Rabbit polyclonal anti-uS15m (dilution: 1:5000)	ProteinTech	Cat# 17006-1-AP
Rabbit polyclonal anti-uL23m (dilution: 1:10 000)	[1]	PRAB1716
Mouse monoclonal anti-FLAG (dilution: 1:1000)	Sigma Prestige	Cat# F1804; Clone# M2
Rabbit polyclonal anti-TIM23 (dilution: 1:1000)	[1]	PRAB1527
Rabbit polyclonal anti-TOM70 (dilution: 1:1000)	[2]	PRAB3280
Mouse monoclonal anti-Calnexin (dilution: 1:500 000)	ProteinTech	Cat# 66903-1-Ig; Clone# 2A2C6
Rabbit polyclonal anti-ND1 (dilution: 1:100 000)	This study*	PRAB5021
Rabbit polyclonal anti-ND2 (dilution: 1:200)	ProteinTech	Cat# 19704-1-AP
Rabbit polyclonal anti-NDUFB8 (dilution: 1:5 000)	[3]	PRA3765
Mouse monoclonal anti-SDHA (dilution: 1:20 000)	Invitrogen	Cat#459200; Clone# 2E3GC12FB2AE2
Rabbit polyclonal anti-CYTB (dilution: 1:1000)	This study*	PRAB5131
Rabbit polyclonal anti-RIESKE (dilution: 1:10 000)	[1]	PRAB1512
Rabbit polyclonal anti-COX1 (dilution: 1:2000)	[1]	PRAB5121
Mouse monoclonal anti-COX2 (dilution: 1:1000)	Abcam	Cat# ab110258; Clone# 12C4F12
Rabbit polyclonal anti-COX4l (dilution: 1:10 000)	[1]	PRAB1522
Rabbit polyclonal anti-ATP5B (dilution: 1:20 000)	[1]	PRAB4826
Rabbit polyclonal anti-ATP6 (dilution: 1:5000)	This study*	PRAB5159
Rabbit polyclonal anti-C12orf62 (dilution: 1:1000)	[1]	PRAB 4845
Rabbit polyclonal anti-MITRAC12 (dilution: 1:1000)	[1]	PRAB3761
Rabbit polyclonal anti-MITRAC15 (dilution: 1:2000)	[2]	PRAB4814
Rabbit polyclonal anti-mtRF1 (dilution: 1:500)	This study	RRDAB5461
Rabbit polyclonal anti-mtRF1a (dilution: 1:1000)	ProteinTech	Cat# 16694-1-AP
Rabbit polyclonal anti-mL62/ICT1 (dilution: 1:1000)	ProteinTech	Cat# 10403-1-AP
Rabbit polyclonal anti-C12ORF65 (dilution: 1:500)	ProteinTech	Cat# 24646-1-AP
Rabbit polyclonal anti-MTRES1 (dilution: 1:1000)	Sigma	Cat# HPA049535
Goat IgG anti-rabbit IgG (H+L)-HRPO (dilution: 1:5000)	dianova	Cat# 111-035-144
Goat IgG anti-mouse IgG (H+L)-HRPO (dilution: 1:5000)	dianova	Cat# 115-035-146
* antibodies were verified as shown in Supplementary Fig. S6		
**Chemicals**		
L-[^{35}S]methionine	Hartmann Analytic	Cat# SCM-01
Adenosine 5'-triphosphate, [γ-^{32}P]	Hartmann-Analytic	Cat# SRP-501
Emetine dihydrochloride hydrate	Sigma-Aldrich	Cat# 219282
Anisomycin	Sigma-Aldrich	Cat# A5862
Lipofectamine 3000	Invitrogen	Cat# L3000-015
GeneJuice	Novagen	Cat# 70967-3
Alt-R® CRISPR-Cas9 tracrRNA, ATTO™ 550	Integrated DNA technologies	Cat# 1075927
Alt-R® S.p. Cas9 Nuclease V3	Integrated DNA technologies	Cat# 1081058
TRIzol® Reagent	Ambion	Cat# 15596018
**Critical Commercial Assays**		
Rapid DNA Ligation Kit	ThermoFisher Scientific	Cat# K1423

T4 Polynucletide Kinase (T4 PNK)	ThermoFisher Scientific	Cat# EK0031
KOD Hot Start DNA Polymerase	Merck	Cat# 71086-3
Wizard® Plus SV Minipreps DNA Purification System	Promega	Cat# A1460
Wizard® SV Gel and PCR Clean-Up System	Promega	Cat# A9282
QuikChange Lightning Site-Directed Mutagenesis Kit	Agilent Technologies	Cat# 210519-5
TOPO TA Cloning® Kit	ThermoFisher Scientific	Cat# 45-0030
OneShot®TOP10 Chemically Competent Cells	ThermoFisher Scientific	Cat# C404004
Complex I Enzyme Activity Assay Kit (Colorimetric)	abcam	Cat# ab109721
Seahorse FluxPaks	Agilent Technologies	Cat# 102416-100
MitoSOX™ Red Mitochondrial Superoxide Indicator	ThermoFisher Scientific	Cat# M36008
**Experimental Models: Cell Lines**		
HEK293-Flp-In T-Rex	ThermoFisher Scientific	R78007
HEK293-Flp-In T-Rex-*mtRF1*$^{-/-}$	This study	N/A
HEK293-Flp-In T-Rex-*mtRF1a*$^{-/-}$	This study	N/A
HEK293-Flp-In T-Rex-mtRF1$^{FLAG\text{-}GGQ}$	This study	N/A
HEK293-Flp-In T-Rex-mtRF1$^{FLAG\text{-}AAQ}$	This study	N/A
HEK293-Flp-In T-Rex-mtRF1a$^{FLAG\text{-}GGQ}$	This study	N/A
HEK293-Flp-In T-Rex-mtRF1a$^{FLAG\text{-}AAQ}$	This study	N/A
**Oligonucleotides**		
Guide RNA: targeting the Exon 2 of mtRF1: 5’-TGTTAAGTAAGAATTGGTCC-3’	This study; IDT	N/A
Guide RNA: targeting the Exon 1 of mtRF1a: 5’-CTCCGGTAGCCCGCCGCTGG-3’	This study; IDT	N/A
siRNA C12orf65 Oligo: GCAAAGGAAACCCUGGAAA	This study; Eurogentec	N/A
Primer: Generation of the FLAG-tagged version of mtRF1 Forward: 5‘-CTCTCCAAGCTTCCACCATGAATCGTCACCTGTGTGTTTGGC-3‘	This study; Microsynth	N/A
Primer: Generation of the FLAG-tagged version of mtRF1 Reverse: 5‘-CTTTCTCTCGAGCTACTTATCGTCGTCATCCTTGTAATCTTTTGCTGATTTAAGGTGTTCATCC-3‘	This study; Microsynth	N/A
Primer: Generation of the FLAG-tagged mutant (GGQ→AAQ) version of mtRF1: Forward: 5’-GATACATTTCGAGCCAAAGGAGCAGCAGCGCAGCATGTTAATAAAAC-3’	This study; Microsynth	N/A
Primer: Generation of the FLAG-tagged mutant (GGQ→AAQ) version of mtRF1 Reverse: 5’-CACTATCAGTTTTATTAACATGCTGCGCTGCTGCTCCTTTGGCTC-3’	This study; Microsynth	N/A

Primer: Generation of the FLAG-tagged version of mtRF1a Forward: 5'-CTCTCCAAGCTTCCACCATGCGGTCCCGGGTTCTGTGGG-3'	This study; Microsynth	N/A
Primer: Generation of the FLAG-tagged version of mtRF1a Reverse: 5'-CTTTCTGATATCCTACTTATCGTCGTCATCCTTGTAATCAACTTTTTGGGAAATAATTTCTACTAAAGATTC-3'	This study; Microsynth	N/A
Primer: Generation of the FLAG-tagged mutant (GGQ→AAQ) version of mtRF1a Forward: 5'-GACACTAAGCGAGCCAGTGGAGCTGCGGCGCAGCATGTAAATAC-3'	This study; Microsynth	N/A
Primer: Generation of the FLAG-tagged mutant GGQ→AAQ) version of mtRF1a Reverse: 5'-CACTGTCCGTGGTATTTACATGCTGCGCCGCAGCTCCACTGGCTC-3'	This study; Microsynth	N/A
Probe: targeting MTRNR1 (12S rRNA) 5'-TCGATTACAGAACAGGCTCCTCTAG-3'	[4]	N/A
Probe for northern blot: targeting MTRNR2 (16S rRNA)5'-GTTTGGCTAAGGTTGTCTGGTAGTA-3'	[4]	N/A
Probe for northern blot: targeting MTCO1 5'- GTCAGTTGCCAAAGCCTCCGATTATG-3'	[4]	N/A
Probe for northern blot: targeting MTCO2 5'-GACGTCCGGGAATTGCATCTGTTTT-3'	[4]	N/A
Probe for northern blot: targeting MTCYTB 5'-CGTGTGAGGGTGGGACTGTCTACTG-3'	This study; Microsynth	N/A
Probe for northern blot: targeting 18S-rRNA 5'-TTTACTTCCTCTAGATAGTCAAGTTCGACC-3'	[5]	N/A
**Recombinant DNA**		
pOG44 Flp-Recombinase Expression Vector	ThermoFisher Scientific	Cat# V600520
pcDNA5/FRT/TO	ThermoFisher Scientific	Cat# V6520-20
**Software and Algorithms**		
ImageJ	[6]	https://imagej.nih.gov/ij/; v.2.1.0
ImageQuant TL	GE Healthcare	v.8.1
Seahorse Wave Desktop	Agilent Technologies	v.2.6.1.53
FACS-Diva software	BD Biosciences	v.9.0.1
nSolver software	NanoString	v.4.0.70

Supplementary Table 2. nCounter tube sequences for NanoString analyses (provided by IDT)

Mito_JCM__008.1:591_T001	CTTCATCAGGGTTTGCTGAAGATGGCGGTATATAGGCTGAGCAAGAGGTGCCTCAAGACCTAAGCGACAGCGTGACCTTGTTTCA
Mito_JCM__009.1:481_T002	CTTTCTTAATTGGTGGCTGCTTTTAGGCCTACTATGGGTGTTAAATTTTTCATCCTCTTCTTTTCTTGGTGTTGAGAAGATGCTC
HSMT_ND1.1:461_T003	GTTCTTGTGTTGTGATAAGGGTGGAGAGGTTAAAGGAGCCACTTATTAGTCACAATTCTGCGGGTTAGCAGGAAGGTTAGGGAAC
HSMT_ND2.1:444_T004	CCTGCTATGATGGATAAGATTGAGAGAGTGAGGAGAAGGCTTACGTTTAGCTGTTGAGATTATTGAGCTTCATCATGACCAGAAG
HSMT_COX1.1:873_T005	GCGGAGGTGAAATATGCTCGTGTGTCTACGTCTATTCCTACTGTAAATATCAAAGACGCCTATCTTCCAGTTTGATCGGGAAACT

HSMT_COX2.1:216_T006	TCGTCTGTTATGTAAAGGATGCGTAGGGATGGGAGGGCGATGAGGACTAGCGAACCTAACTCCTCGCTACATTCCTATTGTTTTC
HSMT_ATP8.1:82_T007	TATTTTTATGGGCTTTGGTGAGGGAGGTAGGTGGTAGTTTGTGTTTAATACCAATTTGGTTTTACTCCCCTCGATTATGCGGAGT
HSMT_ATP6.1:331_T008	GTGGGCTAGGGCATTTTTAATCTTAGAGCGAAAGCCTATAATCACTGTGCCTTTCGGGTTATATCTATCATTTACTTGACACCCT
HSMT_COX3.1:539_T009	ATGTTGAGCCGTAGATGCCGTCGGAAATGGTGAAGGGAGACTCGAAGTACCAACAGCCACTTTTTTTCCAAATTTTGCAAGAGCC
HSMT_ND3.1:135_T010	AAGGTAATAGCTACTAAGAAGAATTTTATGGAGAAAGGGACGCGGGCGGGCACCGTGTGGACGGCAACTCAGAGATAACGCATAT
HSMT_ND4L.1:58_T011	TATTCCTTCTAGGCATAGTAGGGAGGATATGAGGTGTGAGCGATATACTACCTGGAGTTTATGTATTGCCAACGAGTTTGTCTTT
HSMT_ND4.1:967_T012	ACTGTGAGTGCGTTCGTAGTTTGAGTTTGCTAGGCAGAATAGTAATGAGGCAGATAAGGTTGTTATTGTGGAGGATGTTACTACA
HSMT_ND5.1:1147_T013	TGCGGTTTCGATGATGTGGTCTTTGGAGTAGAAACCTGTGAGGAAAGGTACTTCCTTCCTGTGTTCCAGCTACAAACTTAGAAAC
HSMT_ND6.1:191_T014	CCTCAGGATACTCCTCAATAGCCATCGCTGTAGTATATCCAAAGACAACCCATAAAATTGGTTTTGCCTTTCAGCAATTCAACTT
HSMT_CYTB.1:946_T015	GAGGTCTGCGGCTAGGAGTCAATAAAGTGATTGGCTTAGTGGGCGAAATACTGGTCAAGACTTGCATGAGGACCCGCAAATTCCT
NR_003286.2:1640_T016	AGGGCAGGGACTTAATCAACGCAAGCTTATGACCCGCACTTACTGGGAATCTTTCGTTGGGACGCTTGAAGCGCAAGTAGAAAAC
NR_023379.1:8_T017	TGCTTAGCTTCCGAGATCAGACGAGATCGGGCGCGTTCAGGGCCAGCAGACCTGCAATATCAAAGTTATAAGCGCGT
Mito_JCM__008.1:591_ProbeB	CGAAAGCCATGACCTCCGATCACTCTACACCTTGACCTAACGTCTTTACGTGGGTACTTGCGCTTACTTTGTAGC
Mito_JCM__009.1:481_ProbeB	CGAAAGCCATGACCTCCGATCACTCTTCAGTTATATGTTTGGGATTTTTTAGGTAGTGGGTGTTGAGCTTGAACG
HSMT_ND1.1:461_ProbeB	CGAAAGCCATGACCTCCGATCACTCGATAAATCATATTATGGCCAAGGGTCATGATGGCAGGAGTAATCAGAGGT
HSMT_ND2.1:444_ProbeB	CGAAAGCCATGACCTCCGATCACTCAGTATGCTAAGATTTTGCGTAGCTGGGTTTGGTTTAATCCACCTCAACTG
HSMT_COX1.1:873_ProbeB	CGAAAGCCATGACCTCCGATCACTCCGAGTCAGCTAAATACTTTGACGCCGGTGGGGATAGCGATGATTATGGTA
HSMT_COX2.1:216_ProbeB	CGAAAGCCATGACCTCCGATCACTCAGTACCATTGGTGGCCAATTGATTTGATGGTAAGGGAGGGATCGTTGACC
HSMT_ATP8.1:82_ProbeB	CGAAAGCCATGACCTCCGATCACTCAGCGAACAGATTTTCGTTCATTTTGGTTCTCAGGGTTTGTTATAATTTTT
HSMT_ATP6.1:331_ProbeB	CGAAAGCCATGACCTCCGATCACTCATAATAACTAGTATGGGGATAAGGGGTGTAGGTGTGCCTTGTGGTAAGAA
HSMT_COX3.1:539_ProbeB	CGAAAGCCATGACCTCCGATCACTCAGTTGAGCCAATAATGACGTGAAGTCCGTGGAAGCCTGTGGCTACAAAAA
HSMT_ND3.1:135_ProbeB	CGAAAGCCATGACCTCCGATCACTCGGGCTCATGGTAGGGGTAAAAGGAGGGCAATTTCTAGATCAAATAATAAG
HSMT_ND4L.1:58_ProbeB	CGAAAGCCATGACCTCCGATCACTCGAGTGGGTGTTGAGGGTTATGAGAGTAGCTATAATGAACAGCGATAGTAT
HSMT_ND4.1:967_ProbeB	CGAAAGCCATGACCTCCGATCACTCGCTATTAGTGGGAGTAGAGTTTGAAGTCCTTGAGAGAGGATTATGATGCG
HSMT_ND5.1:1147_ProbeB	CGAAAGCCATGACCTCCGATCACTCGTAGCGATGAGAGTAATAGATAGGGCTCAGGCGTTTGTGTATGATATGTT
HSMT_ND6.1:191_ProbeB	CGAAAGCCATGACCTCCGATCACTCCGCTAACCCCACTAAAACACTCACCAAGACCTCAACCCCTGACCCCCATG
HSMT_CYTB.1:946_ProbeB	CGAAAGCCATGACCTCCGATCACTCATGGTAAAAGGGTAGCTTACTGGTTGTCCTCCGATTCAGGTTAGAATGAG
NR_003286.2:1640_ProbeB	CGAAAGCCATGACCTCCGATCACTCGCCTCACTAAACCATCCAATCGGTAGTAGCGACGGGCGGTGTGTACAA
NR_023379.1:8_ProbeB	CGAAAGCCATGACCTCCGATCACTCCGGTCTCCCATCCAAGTACTAACCAGGCCCGACCC

Supplementary References

1. Richter-Dennerlein, R. *et al.* Mitochondrial Protein Synthesis Adapts to Influx of Nuclear-Encoded Protein. *Cell* **167,** 471–483.e10 (2016).
2. Dennerlein, S. *et al.* MITRAC7 Acts as a COX1-Specific Chaperone and Reveals a Checkpoint during Cytochrome c Oxidase Assembly. *Cell Reports* **12,** 1644–1655 (2015).
3. Dennerlein, S. *et al.* Defining the interactome of the human mitochondrial ribosome identifies SMIM4 and TMEM223 as respiratory chain assembly factors. *Elife* **10,** (2021).
4. Lavdovskaia, E. *et al.* Dual function of GTPBP6 in biogenesis and recycling of human mitochondrial ribosomes. *Nucleic Acids Research* **48,** 12929–12942 (2020).
5. Larburu, N. *et al.* Structure of a human pre-40S particle points to a role for RACK1 in the final steps of 18S rRNA processing. *Nucleic Acids Research* **44,** 8465–8478 (2016).
6. Schneider, C. A., Rasband, W. S. & Eliceiri, K. W. NIH Image to ImageJ: 25 years of image analysis. *Nat Methods* **9,** 671–675 (2012).
