# Supplementary material for: Human mtRF1 terminates COX1 translation and its ablation induces mitochondrial ribosome-associated quality control
Source: Nat Commun. 2022 Oct 27;13:6406. doi: 10.1038/s41467-022-34088-w (PMC9613700; doi:10.1038/s41467-022-34088-w)
Supplement: Supplementary file 3 — Reporting Summary [file 41467_2022_34088_MOESM3_ESM.pdf]

nature portfolio

Corresponding author(s): Ricarda Richter-Dennerlein

Last updated by author(s): Oct 5, 2022

Reporting Summary

Nature Portfolio wishes to improve the reproducibility of the work that we publish. This form provides structure for consistency and transparency in reporting. For further information on Nature Portfolio policies, see our Editorial Policies and the Editorial Policy Checklist.

Statistics

For all statistical analyses, confirm that the following items are present in the figure legend, table legend, main text, or Methods section.

n/a	Confirmed	
☐	☒	The exact sample size (*n*) for each experimental group/condition, given as a discrete number and unit of measurement
☐	☒	A statement on whether measurements were taken from distinct samples or whether the same sample was measured repeatedly
☐	☒	The statistical test(s) used AND whether they are one- or two-sided<br>*Only common tests should be described solely by name; describe more complex techniques in the Methods section.*
☒	☐	A description of all covariates tested
☒	☐	A description of any assumptions or corrections, such as tests of normality and adjustment for multiple comparisons
☐	☒	A full description of the statistical parameters including central tendency (e.g. means) or other basic estimates (e.g. regression coefficient) AND variation (e.g. standard deviation) or associated estimates of uncertainty (e.g. confidence intervals)
☐	☒	For null hypothesis testing, the test statistic (e.g. *F*, *t*, *r*) with confidence intervals, effect sizes, degrees of freedom and *P* value noted<br>*Give P values as exact values whenever suitable.*
☒	☐	For Bayesian analysis, information on the choice of priors and Markov chain Monte Carlo settings
☒	☐	For hierarchical and complex designs, identification of the appropriate level for tests and full reporting of outcomes
☒	☐	Estimates of effect sizes (e.g. Cohen's *d*, Pearson's *r*), indicating how they were calculated

Our web collection on statistics for biologists contains articles on many of the points above.

Software and code

Policy information about availability of computer code

Data collection	FACS-Diva software (BD Biosciences; v.9.0.1); nSolver software (nanoString; v.4.0.70); Seahorse Wave Desktop (Agilent Technologies; v.2.6.1.53);
Data analysis	FACS-Diva software (BD Biosciences; v.9.0.1); nSolver software (nanoString; v.4.0.70); Seahorse Wave Desktop (Agilent Technologies; v.2.6.1.53); ImageJ (https://imagej.nih.gov/ij; v2.1.0); ImageQuant TL (GE Healthcare; v.8.1)

For manuscripts utilizing custom algorithms or software that are central to the research but not yet described in published literature, software must be made available to editors and reviewers. We strongly encourage code deposition in a community repository (e.g. GitHub). See the Nature Portfolio guidelines for submitting code & software for further information.

Data

Policy information about availability of data

All manuscripts must include a data availability statement. This statement should provide the following information, where applicable:

- Accession codes, unique identifiers, or web links for publicly available datasets
- A description of any restrictions on data availability
- For clinical datasets or third party data, please ensure that the statement adheres to our policy

We included a data availability statement in a revised version of the manuscript:
Material will be available upon reasonable request and source data are provided with this paper. The original data generated in this study are provided in the

Supplementary Information and the Source Data file, and are available through https://figshare.com/ with the digital object identifier (DOI) 10.6084/m9.figshare.21276411.

Human research participants

Policy information about studies involving human research participants and Sex and Gender in Research.

Reporting on sex and gender	N/A
Population characteristics	N/A
Recruitment	N/A
Ethics oversight	N/A

Note that full information on the approval of the study protocol must also be provided in the manuscript.

Field-specific reporting

Please select the one below that is the best fit for your research. If you are not sure, read the appropriate sections before making your selection.

☒ Life sciences ☐ Behavioural & social sciences ☐ Ecological, evolutionary & environmental sciences

For a reference copy of the document with all sections, see nature.com/documents/nr-reporting-summary-flat.pdf

Life sciences study design

All studies must disclose on these points even when the disclosure is negative.

Sample size	Sample size was determined to demonstrate clear reproducibility of the results (at least three independent biological replicates). When quantification has been applied, sufficient replicates were performed to allow statistical analyses of the data using one-tailed unpaired (equal variances) student's t-test, which requires three replicates.
Data exclusions	We did not exclude data.
Replication	All attempts at replication were successful. The number of replicates of each experiment presented in each figure are given in the figure legend. Experiments have been performed at least three times.
Randomization	Randomization was not relevant to this study as randomization is not applicable during biochemical and cell biological experiments.
Blinding	Blinding was not relevant to this study. Experiments mostly compared specific phenotypes and treatments of otherwise comparable samples. To perform the experiments, it was necessary for the investigators to be aware of the applied conditions.

Reporting for specific materials, systems and methods

We require information from authors about some types of materials, experimental systems and methods used in many studies. Here, indicate whether each material, system or method listed is relevant to your study. If you are not sure if a list item applies to your research, read the appropriate section before selecting a response.

Materials & experimental systems

n/a	Involved in the study	
☐	☒	Antibodies
☐	☒	Eukaryotic cell lines
☒	☐	Palaeontology and archaeology
☒	☐	Animals and other organisms
☒	☐	Clinical data
☒	☐	Dual use research of concern

Methods

n/a	Involved in the study	
☒	☐	ChIP-seq
☐	☒	Flow cytometry
☒	☐	MRI-based neuroimaging

Antibodies

Antibodies used	Rabbit polyclonal anti-uS15m (ProteinTech; Cat# 17006-1-AP); Rabbit polyclonal anti-uL23m (homemade; provided by P.Rehling); Mouse monoclonal anti-FLAG (Sigma Prestige; Cat# F1804; Clone# M2); Rabbit polyclonal anti-TIM23 (homemade; provided by

P.Rehling); Rabbit polyclonal anti-TOM70 (homemade; provided by P.Rehling); Mouse monoclonal anti-Calnexin (ProteinTech; Cat#66903-1-Ig; Clone # 2A2C6); Rabbit polyclonal anti-ND1 (homemade; provided by P.Rehling); Rabbit polyclonal anti-ND2 (ProteinTech; Cat# 19704-1-AP); Rabbit polyclonal anti-NDUFB8 (homemade; provided by P.Rehling); Mouse monoclonal anti-SDHA (Invitrogen; Cat#459200; Clone# 2E3GC12FB2AE2); Rabbit polyclonal anti-CYTB (homemade; provided by P.Rehling); Rabbit polyclonal anti-RIESKE (homemade; provided by P.Rehling); Rabbit polyclonal anti-COX1 (homemade; provided by P.Rehling); Mouse monoclonal anti-COX2 (Abcam; Cat# ab110258; Clone# 12C4F12); Rabbit polyclonal anti-COX4I (homemade; provided by P.Rehling); Rabbit polyclonal anti-ATP5B (homemade; provided by P.Rehling); Rabbit polyclonal anti-ATP6 (homemade; provided by P.Rehling); Rabbit polyclonal anti-C12ORF62 (homemade; provided by P.Rehling); Rabbit polyclonal anti-MITRAC12 (homemade; provided by P.Rehling); Rabbit polyclonal anti-MITRAC15 (homemade; provided by P.Rehling); Rabbit polyclonal anti-mtRF1 (homemade; this study); Rabbit polyclonal anti-mtRF1a (ProteinTech; Cat# 16694-1-AP); Rabbit polyclonal anti-mL62/ICT1 (ProteinTech; Cat# 10403-1-AP); Rabbit polyclonal anti-C12ORF65 (ProteinTech; Cat# 24646-1-AP); Rabbit polyclonal anti-MTRES1 (Sigma; Cat# HPA049535); Goat IgG anti-mouse IgG (H+L)-HRPO (Dianova; Cat#115-035-146; Lot#154319); Goat IgG anti-rabbit IgG (H+L)-HRPO (Dianova; Cat#111-035-144; Lot#161546)

Validation

Antibodies from ProteinTech, Sigma, Invitrogen, Abcam have been validated by the respective company and have been used in previous studies (e.g. Lavdovskaia et al., 2018, 2020). Antibodies provided by Peter Rehling have been validated in previous studies (e.g. Richter-Dennerlein et al. 2016; Wang et al. 2020; Dennerlein et al., 2015). Antibodies against mtRF1a and mtRF1 have been validated by western blotting using generated human knockout and RFs-overexpressing cell lines (e.g. Fig. 1b.; 3d). Antibodies against CYTB, ND1 and ATP6 have been validated using 143B-Rho0 cells (Supplementary Fig. 6).

Eukaryotic cell lines

Policy information about cell lines and Sex and Gender in Research

Cell line source(s)

HEK293-Flp-In T-Rex (Thermo Fisher Scientific; R78007); CRISPR-Cas9 generated knockout cell lines HEK293-Flp-In T-Rex-mtRF1-/- and HEK293-Flp-In T-Rex-mtRF1a-/-; Rescue and mutant cell lines were generated from the respective knockout cell lines: HEK293-Flp-In T-Rex-mtRF1FLAG-GGQ; HEK293-Flp-In T-Rex-mtRF1FLAG-AAQ; HEK293-Flp-In T-Rex-mtRF1aFLAG-GGQ; HEK293-Flp-In T-Rex-mtRF1aFLAG-AAQ

Authentication

Used HEK293-Flp-In T-Rex cell lines, containing blasticidine resistance locus, were routinely treated with blasticidine S to ensure authenticity of used cell lines. Knockout cell lines were confirmed by western blotting and gDNA sequencing. Rescue and mutant cell lines were confirmed by western blotting.

Mycoplasma contamination

Cells were systematically confirmed to be negative for the presence of Mycoplasma by GATC Biotech.

Commonly misidentified lines
(See ICLAC register)

No commonly misidentified cell lines were used.

Flow Cytometry

Plots

Confirm that:

☒ The axis labels state the marker and fluorochrome used (e.g. CD4-FITC).

☒ The axis scales are clearly visible. Include numbers along axes only for bottom left plot of group (a 'group' is an analysis of identical markers).

☐ All plots are contour plots with outliers or pseudocolor plots.

☒ A numerical value for number of cells or percentage (with statistics) is provided.

Methodology

Sample preparation

HEK293-Flp-In T-Rex (Thermo Fisher Scientific) were co-transfected with crRNA-tracrRNA-ATTO550 duplex and Cas9 nuclease according to the manufacturers instructions and single cells were sorted using FACS using BD FACS Canto II.
For analysis of ROS, either 10^6 HEK293-Flp-In T-Rex WT or KO cells were stained with 5 µM MitoSOX Red (invitrogen) for 10 min at 37 °C, washed 1x in PBS and analyzed using BD LSR Fortessa X20.

Instrument

BD FACS Canto II (Becton Dickinson) & BD LSR Fortessa X20

Software

FACS-Diva software

Cell population abundance

For sorting cells transfected with CRISPR/Cas9-technology, the main cell population was determined by excluding debris or death cells using FSC/SSC and further refined by excluding cell doublets via the FSC-A-/FSC-W ratio and further using SSC-A/SSC-W ratio.
For ROS analysis, the main cell population was determined by excluding debris or death cells using FSC/SSC and further refined by excluding cell doublets via the SSC-A/SSC-W ratio. Approx. 50-60 % of 10 000 gated cells were further analyzed.

Gating strategy

To discriminate between positive and negative cells, un-transfected or untreated cells served as a negative control to define the respective gates.

☒ Tick this box to confirm that a figure exemplifying the gating strategy is provided in the Supplementary Information.
